# Editorial for the Special Issue on Electronics for Sensors II

**DOI:** 10.3390/s23031640

**Published:** 2023-02-02

**Authors:** Giuseppe Ferri, Gianluca Barile, Alfiero Leoni

**Affiliations:** Department of Industrial and Information Engineering and Economics, University of L’Aquila, 67100 L’Aquila, Italy

Sensor signals are physical, chemical, or biological quantities that evolve over time. Sensor systems contain one or more sensors and an electronic circuit (named an interface or read-out) that converts the input signals from the sensor into output signals suitable for correct reading and quantification of physical, chemical, or biological parameters (or variations thereupon) in an electric/electronic form [1].

The suitable design of these sensor interfaces has always played a fundamental role in improving the characteristics of the whole sensor system, so it is very important to pay the utmost attention to this task, especially in light of the problems related to technology scaling and different technology integrations. Moreover, research on circuits and systems for interfacing sensors (in particular, resistive and capacitive sensors [2,3,4]) has become, and will surely remain, a highly prioritized, widespread, and lively topic. This is because any real-world application has inevitably to sense and manipulate some kind of magnitude.

Modern interfaces must satisfy parameters strictly related to the magnitude under evaluation, such as sensitivity and resolution, in addition to fulfilling new constraints from the particular macro-system into which the interface is embedded, e.g., low power consumption and low cost. To make this even more challenging, with the first interfacing stage typically analog, it gains few benefits from technology scaling in terms of chip area reduction [5] and power consumption, whereas it typically has to contend with parasitic elements of the same magnitude as the sensing element.

This Special Issue gathers a number of works that present new possible solutions regarding electronics for sensors, both related to the analog processing stage and the digital processing section, in addition to modern applications. It contains a selection of papers coming from many different research groups in different countries.

In [6], Y. C. Lee and V. A. Hoang developed a wireless sensor system (WSS) for real-time monitoring of shaft positioning in marine applications. Specifically, the WSS is battery free, since it embeds an inductive wireless power transfer module that supplies power to the sensor. The authors aim to use the proposed sensor in smart/automated ships [7,8,9,10,11].

In [12], C. Qin et al. introduced an energy-efficient BJT-based temperature sensor. In the proposed manuscript, the authors show both the analog front-end, as well as the Σ/Δ ADC converter employed to provide a digital output for the user. The temperature sensor contains a cascoded floating inverter as an integrating amplifier rather than simply as a transconductance amplifier, allowing to reduce power consumption. Additionally, to improve read-out accuracy, the authors designed the sensor to make use of compensation techniques such as dynamic element matching and chopping [13,14,15].

In [16], R. Riem, J. Raman, and P. Rombouts proposed a high bandwidth, low-offset Hall plate read-out system with a randomized four-phase spinning-current scheme. In the manuscript, they demonstrate how it is possible to reduce inessential components to minimize the offset parameter. In conjunction with this, the authors designed the system to embed spread-spectrum mode offset-reduction loops, enabling a very large notch-free bandwidth to be maintained and further reducing error sources [17,18,19].

In [20], S. Pettinato et Al. designed an electronic sensing system to accurately measure very narrow pulsed-current signals. Specifically, the authors described how it is possible to utilize a synchronous demodulation technique to increase improved performances in terms of signal-to-noise ratio compared to conventional free-running systems [21,22,23].

In [24], L. Safari et Al. proposed a review manuscript about second generation voltage conveyor (VCII)-based analog electronic interfaces for bioelectrical signals. In the manuscript, the authors reported the working principles of the VCII and a wide number of applications available from the literature in the fields of current-mode Wheatstone bridges (CMWBs), silicon photomultipliers, ultrasonic sensors, differential capacitive sensors and ECG/EEG applications [25,26,27,28,29].

Interest in wearable sensors is on the rise [30,31], and several interesting papers have been presented recently. In [32], D. Asiain et Al. introduced a multi-sensor wearable headband, a new physiological signal acquisition multi-sensory platform for emotion identification (MsWH). The proposed system offers several sensors to measure human physiological signals, such as skin temperature, blood oxygen saturation, heart rate (and its variation), movement/position of the user, and electrodermal activity/bioimpedance, together with a camera that can track human facial movements, so the viewing area remains constant. The whole system design is covered in this work, starting from the conditioning electronics to the microprocessor unit and data sending. In [33], H. Zhang et Al. proposed a method to reduce the measurement error in sensor arrays for wearable electronics based on a dynamic zero current technique that showed fewer errors than other methods, such as the zero potential technique [34,35].

Digital sensor acquisition and data processing is also a pervasive field, as digital electronics is spreading within sensor systems, increasing the possibilities and data analysis [36,37]. In [38], R. Esteve Bosch et Al. proposed a new data compression algorithm in an FPGA-based data acquisition system for the NEXT-100 collaboration detectors. The system is equipped with a large number of Photomultiplier Tubes (PMTs) and Silicon Photomultipliers (SiPM) for energy measurement, with an expected data throughput of over 900 MB/s. As for this huge data rate, the proposed data compression algorithm reduces the throughput and improves the overall functionality. In [39], A. Tsukahara et Al. presented a processor implemented in FPGA hardware to perform Capacitive-coupling Impedance Spectroscopy (CIS) that can provide the full acquisition and processing sequence in a few milliseconds.

Finally, biosensors and electrochemical sensors are strongly considered for plant monitoring and energy harvesting in the emerging agriculture 4.0 field, which is establishing the basis for future farming technology [40,41,42]. In [43], D. Wang et Al. reported a study with the aim of assessing the usage of electrochemical sensors for the identification of different plant species, resulting in a high level of effectiveness that is increased if different pattern recognition techniques are used.

## References

[1] Falconi C., Martinelli E., Di Natale C., D’Amico A., Maloberti F., Malcovati P., Baschirotto A., Stornelli V., Ferri G. (2007). Electronic Interfaces. Sens. Actuators B Chem..

[2] Baschirotto A., Capone S., D’Amico S., Di Natale C., Ferragina V., Ferri G., Francioso L., Grassi M., Guerrini N., Malcovati P. (2008). A portable integrated wide-range gas sensing system with smart A/D front-end. Sens. Actuators B.

[3] Wilson D.M. (2022). Electronic Interface Circuits for Resistance-Based Sensors. IEEE Sens. J..

[4] Somappa L., Malik S., Aeron S., Sonkusale S., Baghini M.S. (2021). High Resolution Frequency Measurement Techniques for Relaxation Oscillator Based Capacitive Sensors. IEEE Sens. J..

[5] Ferri G., Pennisi S. A 1.5-V current-mode capacitance multiplier. Proceedings of the Tenth ICM (International Conference on Microelectronics—Cat. No.98EX186).

[6] Lee Y.C., Hoang V.A. (2023). Battery-Free and Real-Time Wireless Sensor System on Marine Propulsion Shaft Using a Wireless Power Transfer Module. Sensors.

[7] Reda K., Yan Y. (2019). Vibration Measurement of an Unbalanced Metallic Shaft Using Electrostatic Sensors. IEEE Trans. Instrum. Meas..

[8] Xia Q., Yan L. (2016). Application of Wireless Power Transfer Technologies and Intermittent Energy Harvesting for Wireless Sensors in Rotating Machines. Wirel. Power Transf..

[9] Leoni A., Ulisse I., Pantoli L., Errico V., Ricci M., Orengo G., Giannini F., Saggio G. (2019). Energy Harvesting Optimization for Built-in Power Replacement of Electronic Multisensory Architecture. AEU—Int. J. Electron. Commun..

[10] Ragnoli M., Barile G., Leoni A., Ferri G., Stornelli V. (2020). An Autonomous Low-Power LoRa-Based Flood-Monitoring System. J. Low Power Electron. Appl..

[11] Fusacchia P., Muttillo M., Leoni A., Pantoli L., Parente F.R., Stornelli V., Ferri G. (2016). A Low Cost Fully Integrable in a Standard CMOS Technology Portable System for the Assessment of Wind Conditions. Procedia Eng..

[12] Qin C., Huang Z., Liu Y., Li J., Lin L., Tan N., Yu X. (2022). An Energy-Efficient BJT-Based Temperature Sensor with ±0.8 °C (3σ) Inaccuracy from −50 to 150 °C. Sensors.

[13] Souri K., Souri K., Makinwa K. A 40 µW CMOS Temperature Sensor with an Inaccuracy of ±0.4 °C (3σ) from −55 °C to 200 °C. Proceedings of the ESSCIRC (ESSCIRC).

[14] Yousefzadeh B., Makinwa K.A.A. (2020). A BJT-Based Temperature-to-Digital Converter with a ±0.25 °C 3 σ—Inaccuracy from −40 °C to +180 °C Using Heater-Assisted Voltage Calibration. IEEE J. Solid-State Circuits.

[15] Markus J., Silva J., Temes G.C. (2004). Theory and Applications of Incremental /Spl Delta//Spl Sigma/ Converters. IEEE Trans. Circuits Syst. I Regul. Pap..

[16] Riem R., Raman J., Rombouts P. (2022). A 2 MS/s Full Bandwidth Hall System with Low Offset Enabled by Randomized Spinning. Sensors.

[17] Jiang J., Makinwa K.A.A. (2017). Multipath Wide-Bandwidth CMOS Magnetic Sensors. IEEE J. Solid-State Circuits.

[18] Baltes H.P., Popovic R.S. (1986). Integrated Semiconductor Magnetic Field Sensors. Proc. IEEE.

[19] Munter P.J.A. (1990). A Low-Offset Spinning-Current Hall Plate. Sens. Actuators A Phys..

[20] Pettinato S., Girolami M., Rossi M.C., Salvatori S. (2022). Accurate Signal Conditioning for Pulsed-Current Synchronous Measurements. Sensors.

[21] Ruppert M.G., Harcombe D.M., Ragazzon M.R.P., Moheimani S.O.R., Fleming A.J. (2017). A Review of Demodulation Techniques for Amplitude-Modulation Atomic Force Microscopy. Beilstein J. Nanotechnol..

[22] Wu L., Zhao G., Feng Z. (2022). A High Resolution Synchronous Demodulation Method Based on Gated Integrator for Precision Sensors. IEEE Trans. Instrum. Meas..

[23] Pettinato S., Orsini A., Girolami M., Trucchi D.M., Rossi M.C., Salvatori S. (2019). A High-Precision Gated Integrator for Repetitive Pulsed Signals Acquisition. Electronics.

[24] Safari L., Barile G., Stornelli V., Ferri G. (2019). An Overview on the Second Generation Voltage Conveyor: Features, Design and Applications. IEEE Trans. Circuits Syst. II Express Briefs.

[25] Barile G., Leoni A., Pantoli L., Safari L., Stornelli V. A New VCII Based Low-Power Low-Voltage Front-End for Silicon Photomultipliers. Proceedings of the 2018 3rd International Conference on Smart and Sustainable Technologies (SpliTech).

[26] Safari L., Barile G., Stornelli V., Ferri G., Leoni A. New Current Mode Wheatstone Bridge Topologies with Intrinsic Linearity. Proceedings of the 2018 14th Conference on Ph.D. Research in Microelectronics and Electronics (PRIME).

[27] Barile G., Safari L., Ferri G., Stornelli V. (2019). A VCII-Based Stray Insensitive Analog Interface for Differential Capacitance Sensors. Sensors.

[28] Safari L., Barile G., Stornelli V., Ferri G. (2020). A New Versatile Full Wave Rectifier Using Voltage Conveyors. AEU—Int. J. Electron. Commun..

[29] Stornelli V., Safari L., Barile G., Ferri G. (2021). A New VCII Based Grounded Positive/Negative Capacitance Multiplier. AEU—Int. J. Electron. Commun..

[30] Bonato P. (2003). Wearable Sensors/Systemsand Their Impact onBiomedical Engineering. IEEE Eng. Med. Biol. Mag..

[31] Channa A., Popescu N., Skibinska J., Burget R. (2021). The rise of wearable devices during the COVID-19 pandemic: A systematic review. Sensors.

[32] Asiain D., Ponce de León J., Beltrán J.R. (2022). MsWH: A Multi-Sensory Hardware Platform for Capturing and Analyzing Physiological Emotional Signals. Sensors.

[33] Zhang H., Teoh J.C., Wu J., Yu L., Lim C.T. (2023). Dynamic Zero Current Method to Reduce Measurement Error in Low Value Resistive Sensor Array for Wearable Electronics. Sensors.

[34] Wu J., He S., Li J., Song A. (2016). Cable crosstalk suppression with two-wire voltage feedback method for resistive sensor array. Sensors.

[35] Lazzarini R., Magni R., Dario P. A tactile array sensor layered in an artificial skin. Proceedings of the 1995 IEEE/RSJ International Conference on Intelligent Robots and Systems: Human Robot Interaction and Cooperative Robots.

[36] Leoni A., Esposito P., Stornelli V., Saggio G., Ferri G. (2021). On the use of field programmable gate arrays in light detection and ranging systems. Rev. Sci. Instrum..

[37] Xie Y., Majoros T., Oniga S. (2022). FPGA-Based Hardware Accelerator on Portable Equipment for EEG Signal Patterns Recognition. Electronics.

[38] Esteve Bosch R., Rodríguez Ponce J., Simón Estévez A., Benlloch Rodríguez J.M., Herrero Bosch V., Toledo Alarcón J.F. (2022). Data Compression in the NEXT-100 Data Acquisition System. Sensors.

[39] Tsukahara A., Yamaguchi T., Tanaka Y., Ueno A. (2022). FPGA-Based Processor for Continual Capacitive-Coupling Impedance Spectroscopy and Circuit Parameter Estimation. Sensors.

[40] Leoni A., Ferri G., Ursini D., Zompanti A., Sabatini A., Stornelli V. Towards smart sensor systems for precision farming: Electrode potential energy harvesting from plants’ soil. Proceedings of the 2022 29th IEEE International Conference on Electronics, Circuits and Systems (ICECS).

[41] Sabatini A., Leoni A., Goncalves G., Zompanti A., Marchetta M.V., Cardoso P., Grasso S., Di Loreto M.V., Lodato F., Cenerini C. (2022). Microsystem nodes for soil monitoring via an energy mapping network: A proof-of-concept preliminary study. Micromachines.

[42] Leoni A., Ferri G., Colaiuda D., Stornelli V. Micro energy harvesting from the soil of indoor living plants. Proceedings of the 2022 7th International Conference on Smart and Sustainable Technologies (SpliTech).

[43] Wang D., Li D., Fu L., Zheng Y., Gu Y., Chen F., Zhao S. (2021). Can Electrochemical Sensors Be Used for Identification and Phylogenetic Studies in Lamiaceae?. Sensors.

